# Cationized Hemp Fiber to Improve the Interfacial Adhesion in PLA Composite

**DOI:** 10.3390/polym17050652

**Published:** 2025-02-28

**Authors:** Meylí Valin Fernández, Matías Angelo Monsalves Rodríguez, Carlos Andrés Medina Muñoz, Daniel A. Palacio, Angelo Giovanni Oñate Soto, José Luis Valin Rivera, Francisco Rolando Valenzuela Diaz

**Affiliations:** 1 Department of Mechanical Engineering (DIM), Faculty of Engineering (FI), University of Concepción, Concepción 4070409, Chile; mvalin@udec.cl (M.V.F.); mmonsalves2018@udec.cl (M.A.M.R.); cmedinam@udec.cl (C.A.M.M.); 2 Department of Polymers, Faculty of Chemical Sciences, University of Concepción, Concepción 4070371, Chile; dapalacio@udec.cl; 3 Department of Materials Engineering, Faculty of Engineering (FI), University of Concepción, Concepción 4070415, Chile; aonates@udec.cl; 4 Escuela de Ingeniería Mecánica, Pontificia Universidad Católica de Valparaíso, Valparaíso 2340000, Chile; 5 Department of Materials Engineering and Metallurgy, University of São Paulo, São Paulo 05508-220, Brazil; frrvdiaz@usp.br

**Keywords:** 3D printing, polylactic acid (PLA), hemp fiber, cationization treatment, mechanical properties

## Abstract

3D printing with biodegradable polymers such as polylactic acid (PLA) is a sustainable alternative to conventional petroleum-derived plastics. However, improving the mechanical properties of PLA remains a challenge. This study explores the incorporation of chemically treated hemp fibers to improve the interfacial adhesion and mechanical strength of PLA filaments. Samples with PLA and hemp were prepared by subjecting the fibers to cationization treatment with (3-chloro-hydroxypropyl) tri-methylammonium (EPTA) and functionalization with glycidyl methacrylate (GMA). EPTA improves adhesion mainly through surface modification, increasing reactive functional groups in cellulose, while GMA improves interfacial adhesion by forming covalent bonds with both the fiber and PLA and improves the dispersion of the fiber in the matrix. Mechanical properties were evaluated by tensile testing, as well as fracture morphology by scanning electron microscopy (SEM) and X-ray energy dispersive analysis (EDS). The results showed that the addition of untreated hemp significantly reduced the strength of PLA, but cationization with EPTA improved interfacial adhesion and increased tensile strength by 615%. The combination of treated fibers and GMA further optimized the mechanical properties, reaching values similar to pure PLA. These findings indicate that the chemical modification of natural fibers facilitates their integration into PLA filaments for 3D printing, promoting sustainable materials without compromising mechanical performance.

## 1. Introduction

3D printing is a process that allows manufacturing parts and elements of complex geometry without surplus material, which considerably reduces waste [1]. Currently, most users prefer traditional plastics such as Acrylonitrile Butadiene Styrene (ABS), Acrylonitrile Styrene (ASA), Polyethylene Terephthalate (PET), and Polyethylene Terephthalate Glycol (PETG), which are more harmful to the environment.

Polylactic acid (PLA) is one of the most widely used materials in 3D printing, especially in technologies such as FDM (Fused Deposition Modeling). Its popularity is due to a combination of physical, chemical, environmental and economic properties that make it ideal for a wide range of applications. It is considered one of the easiest materials to print compared to other filaments such as ABS or PETG. This is due to its low extrusion temperature which reduces nozzle wear and minimizes the need for printers with advanced hotends [2]; it does not require a heated bed, although it can improve adhesion if used at temperatures of 50–60 °C, low thermal shrinkage, which minimizes warping and allows large objects to be printed without distortion and good adhesion between layers, reducing defects such as delamination [3]. It is produced from natural sources such as corn starch, sugar cane or beetroot. Although it is not completely biodegradable under standard environmental conditions, it degrades in industrial composting under controlled temperature and humidity. Its production emits less CO_2_ compared to plastics such as ABS, making it more environmentally friendly [4]. Unlike other materials, PLA does not emit toxic fumes during printing, making it suitable for closed spaces and ideal for use in homes, and in some formulations, it can be used in packaging and utensils. It is ideal for aesthetic and detailed parts due to its print quality, thanks to its good fluidity and adhesion between layers. Although it is not the most resistant material in terms of impact or bending, it has good mechanical resistance for applications not subjected to great stress. It is one of the most economical filaments, compared to other materials such as ABS or PETG, and widely available on the market [5].

Some eco-friendly composite filaments have been manufactured incorporating recycled cellulose and rubber-based materials, resulting in 3D printed parts with improved stiffness, toughness and distortion. In this paper, Zander [6] analyzed current trends in the use of recycled plastics in polymer additive manufacturing based on extrusion of materials, presenting a perspective for the future of such materials.

Other authors such as Ghabezi et al. [7] have investigated how to overcome printing and processing challenges, in this case of waste polypropylene (PP) reinforced with short basalt fibers, transforming them into recycled raw material for additive manufacturing. The author used an optimized method to produce filaments and 3D print with these materials, addressing common problems such as adhesion and deformation through innovative techniques while maintaining maximum mechanical performance in the resulting composites. While Sam-Daliri et al. [8] investigated the effect of glass fiber weight fraction on the printability and mechanical properties of filaments and printed samples. The filaments were manufactured by optimizing parameters of the filament extrusion process obtaining a tensile strength of 112 MPa for the filament with a fiber weight content of 40%.

In the first studies carried out to improve the properties of PLA-based filaments, organic compounds such as wheat, rice, bamboo, kenaf and hemp were used [9]. Silva et al. [10] carried out an investigation of the use of hemp fibers cut to an average size of 7 to 10 mm, where the hemp fibers were washed with distilled water and dried in oven at 80 °C for 4 h, during this process a double screw extruder was used to make a double extrusion plus injection process, together with a mold to generate the specimens. These specimens had a weight ratio of 25% hemp fiber and 75% PLA, in which no compatibilizers or further treatments were used. The combination of PLA and hemp fiber made by injection-extrusion method increased tensile, impact and flexural strengths by 2.19%, 44.38% and 16.68% respectively compared to PLA.

Skuratowicz [11] carried out the preparation of a filament based PLA and powder hemp with 75 to 425 μm. Hemp and PLA were added to an extruder in different proportions, one of 5% and another of 10% hemp. From the obtained filament, specimens were manufactured by 3D printing to compare their mechanical properties. The 5% and 10% hemp-reinforced PLA were printed with a nozzle temperature of 200 °C and the bed temperature was set to 60 °C. To maximize the possibility of good bed adhesion, the print speed was set to 60 mm/s, and the first layer speed was set to 10 mm/s. The tensile strength decreased for the specimens with 5% and 10% hemp by 12.14% and 48.89% compared to the PLA specimens, while the tensile elastic modulus increased by 36.49% and 9.32% respectively. The decrease in the tensile strength is mostly due to the hemp particles having a low length diameter ratio (L/D). A good adherence between PLA matrix and fibers requires a higher L/D, that would increase tensile strength and the load could be better distributed through the polymer matrix to the fibers [11,12]. According to the Skuratowicz, another factor that could cause a decrease in tensile strength was the appearance of gaps between the infill lines. These gaps were formed by the lack of sufficient filament during printing since as more hemp is added to the PLA it begins to impede the flow of the polymer, causing slower extrusion favoring the formation of gaps.

The process of modifying natural fiber in terms of size could increase the dispersion of kenaf fiber in the polymer matrix and increase the adhesion bond between the fiber and the matrix of the composites, subsequently improving the interfacial bond between these two phases. According to the study conducted by Jamadi et al. [13] a fiber size ≤100 μm presents better tensile and flexural strength, compared to other sizes. Thus, it is concluded that by reducing the fiber size, the composites could develop high strength performance for industrial applications [13].

The main challenge for hemp to function as a viable filler where its properties are equal or better than those of PLA, at a low economic cost and with good biodegradability lies first in achieving an optimal length in the hemp fiber, knowing the optimal percentages of hemp and PLA, the treatments for the hemp fiber and finally the functionalization of the filament. Studies have addressed the need to improve the interfacial bond between hemp and PLA in order to achieve better adhesion and a more uniform distribution of the fibers in the PLA matrix [14,15,16,17,18].

Khan et al. [19] crushed hemp to sizes ranging from 44 to 400 μm. To improve interface compatibility, they used glycidyl methacrylate (GMA) and tert-butyl per benzoate (TBPB), an initiator for the grafting reaction of GMA onto PLA, achieving better interfacial adhesion between hemp and GMA-grafted PLA. Methacrylate has the property of grafting onto both PLA and hemp and also contains an epoxy group that is capable of reacting with hydroxyl, carboxyl and amine groups. The results obtained from the mechanical properties showed an improvement in impact strength of 3% and flexural elastic modulus of 21.96% compared to PLA.

Xiao et al. [20] made use of ground hemp with an average size of 50 μm in order to activate the hydroxyl groups (OH). This study used as a reference the previous one by Khan et al. [19], using GMA and TBPB as compatibilizer, but adding a toughening agent PBAT to improve the mechanical properties of the filament. The analysis of the behavior of the mechanical properties reflected that the tensile strength increased by 6.67%, the flexural strength increased by 6.06% and the flexural elastic modulus increased by 35.74 by injection-molding method, compared to PLA.

The implementation of biomass for Fused Deposition Modeling (FDM) 3D printing is less dangerous and potentially more cost-effective compared to traditional petrochemical plastics. The development of thermally and mechanically sustainable composites through PLA and natural fibers derived from biomass represents an improvement in costs and biodegradability [21]. The use of hemp fiber for these composites in FDM 3D printing presents very few works in the literature; taking into account that the production of the hemp plant in a country like Chile is 214,000 tons per year [22], the use of hemp fibers for the manufacture of filament can be beneficial if it can be supported. On the other hand, the interfacial incompatibility between hemp fiber and PLA thermoplastic causes weak adhesion at the interface, leading to inadequate mechanical properties.

The present work arises from the need to develop sustainable materials that reduce the environmental impact of manufacturing processes, in line with current sustainability demands. In this context, the combination of PLA, a widely used biodegradable polymer, with hemp fibers, a renewable, low-cost, and highly available resource, offers a promising approach for 3D printing. However, limitations in the interfacial compatibility between these materials represent a critical challenge to achieving optimal mechanical properties. This study addresses this problem by developing innovative treatments, such as the cationization of hemp fibers and the functionalization of the composite with compatibilizers, seeking to improve adhesion at the PLA–hemp interface. The expected results not only seek to contribute to the advancement of composite materials in 3D printing but also to encourage their adoption in sustainable and environmentally responsible industrial applications.

## 2. Materials and Methods

### 2.1. Materials

Virgin PLA, which has an approximate density of 1.24 g/cm^3^, was purchased from Cicla 3D company (Concepción, Chile). The hemp was purchased from a local store in the city of Concepción, in braided jute format with a length of approximately 2 m. For the washing and alkaline activation of the OH groups, sodium hydroxide (NaOH, 99.9%) was used, purchased from Merck company (Darmstadt, Germany), in lentil format. To neutralize the alkaline activation, acetic acid (CH3COOH, 99.8%) was used, also purchased from Merck. For the cationization treatment of hemp activation of the OH groups in the hemp, (3-Chloro-hydroxypropyl) trimethylammonium chloride solution (EPTA, 60 wt% in water) was used, purchased from Merck. Distilled water and ethanol were used to remove the remains of EPTA. Glycidyl methacrylate (GMA, 97.0%) was used to act as a graft between PLA and hemp in order to improve the interfacial bond between the two.

### 2.2. Fiber Grinding

To improve the adhesion of the hemp fiber to the PLA, a fiber grinding process was performed. For this, the Cyclotec 923 mill from the FOSS Analytical company (Buenos Aires, Argentina) was used. The fiber was crushed and separated by the mill filter at an approximate scale of 400–600 μm and at 4 g/s of speed, up to a sample size of approximately 340 g. Since hemp cellulose is composed of bound glucose that contains OH groups in its structure, grinding the hemp helps with the activation of the OH groups. The mechanical process of grinding can break bonds between cellulose molecules, exposing OH groups on the surface of the hemp.

### 2.3. Fiber Treatment

For the hemp fiber washing process, the first step was to immerse the ground fiber in distilled water until it was completely covered and stir for approximately 30 min [20]. Then, the first filtering process was carried out with a Kitasato flask (from Merck company) for approximately 24 h. Once the filtering process was finished, a solution of one liter of 5% NaOH was prepared in a 1 L volumetric flask. The filtered hemp together with the NaOH solution was poured into a container until the fiber was completely covered for 48 h. Then, a second filtering process was carried out that took approximately another 24 h to subsequently carry out a wash to eliminate any excess product. A third filtering process was carried out to eliminate the distilled water, and then a 1% acetic acid solution was made in a 1 L volumetric flask to neutralize the pH of the NaOH. The fiber was filtered for the last time and then left to dry in oven at 35 °C for 48 h to obtain the hemp powder.

A cationization treatment was performed using 5% NaOH solution, which was heated in a three-necked flask on a magnetic stirrer. Three samples (M1, M2, and M3) were prepared by adding 1 g of hemp and stirring at 1500 rpm for 30 min at a temperature of 65 °C. A fourth sample (M4) was also prepared by adding 1 g of hemp and stirring at 1500 rpm for 30 min, but this time at a temperature of 90 °C. Different amounts of EPTA were added to each sample to obtain mixtures with different concentrations, as presented in Table 1. These values were calculated for a concentration of 60%. After this, the samples were stirred for 24 h and then washed with distilled water until reaching a neutral pH. Finally, the EPTA was neutralized with ethanol until any residue was eliminated.

**Figure 1:**
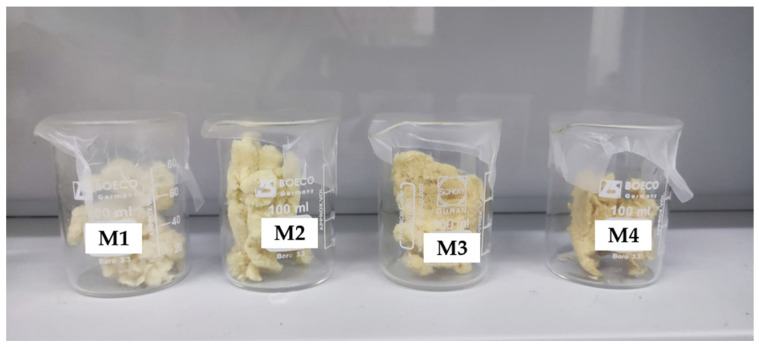
Hemp samples treated with EPTA.

The hemp samples obtained in each experiment are shown in Figure 1, where a variation in their color can be seen. The last step with the hemp fiber obtained is to dry it in the oven for at least 2 days to eliminate moisture. Once removed from the oven, the samples were weighed, and a variation in their weight of approximately 15% was observed.

### 2.4. Mixin Proportions

The specimens produced have the compositions presented in Table 2, which allow studying combinations of only PLA; hemp and PLA; treated hemp and PLA; and treated hemp, compatibilizer, and PLA. It was also considered that the specimens have a proportion of 5% hemp and 95% PLA.

### 2.5. Preparation of the Specimens

**Figure 2:**
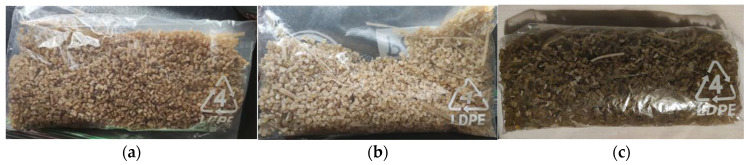
Pellets produced: (**a**) PLA, hemp, and GMA; (**b**) PLA and treated hemp; and (**c**) PLA with treated hemp and GMA.

For the fabrication of the specimens, a Double-Screw Extruder, Model N°BP-8177-ZB from Baopin Technology (Dongguan City, Guangdong Province, China.) was used. The temperatures varied between 140 °C in the stroke and 150 °C at the nozzle. The output speed was regulated according to the filament flow leaving the extruder, which was cooled in water and turned into pellets at a variable speed adapted to the output flow. Figure 2 shows the pellet obtained for some of the configurations shown in Table 2.

The pellet obtained was placed in the manual injector model 150A from the company LNS Technologies (Yucaipa, CA, USA) at 150 °C, the same temperature as the extruder nozzle. Once melted, it was injected into the mold previously heated to 150 °C for 15 min and then left to cool in water until it reached room temperature. The specimens used for the tensile test comply with ASTM D638 and have the design of a V-type beta test specimen.

### 2.6. Tensile Test

Tensile tests were performed on a Zwick Roell ProLine Z005) universal testing machine from Zwick Roell company (Santiago, Chile) according to ASTM D638 [23]. The test speed used was 50 ± 10% mm/min, which is indicated by the standard for non-rigid materials. The objective of the test is to evaluate the efficiency of the hemp treatment and the compatibilizer in their mixture with PLA.

### 2.7. SEM and EDS Analyses

For the electron microscopy analysis, the scanning electron microscope (SEM) Carl Zeiss Gemini SEM 360, model Gemini SEM 360 from the manufacturer Carl Zeiss Microscopy (Madrid, Spain), was used. For the X-ray energy dispersion analysis (EDS), an Oxford Instruments Ultim Max 65 energy dispersion X-ray detector (EDS/EDX) from the manufacturer Carl Zeiss Microscopy (Madrid, Spain), was used, which is coupled to the aforementioned electron microscope.

For the SEM analysis, the sample was placed on a support called a pin stub, and a double-sided adhesive disc with carbon was placed on it. Subsequently, the samples were covered with a conductive material; in this way, the conductivity of the sample was improved, the thermal damage was reduced, and the emission of secondary electrons was improved, which improved the image quality.

## 3. Results

### 3.1. Influence of EPTA on Mixtures

**Figure 3:**
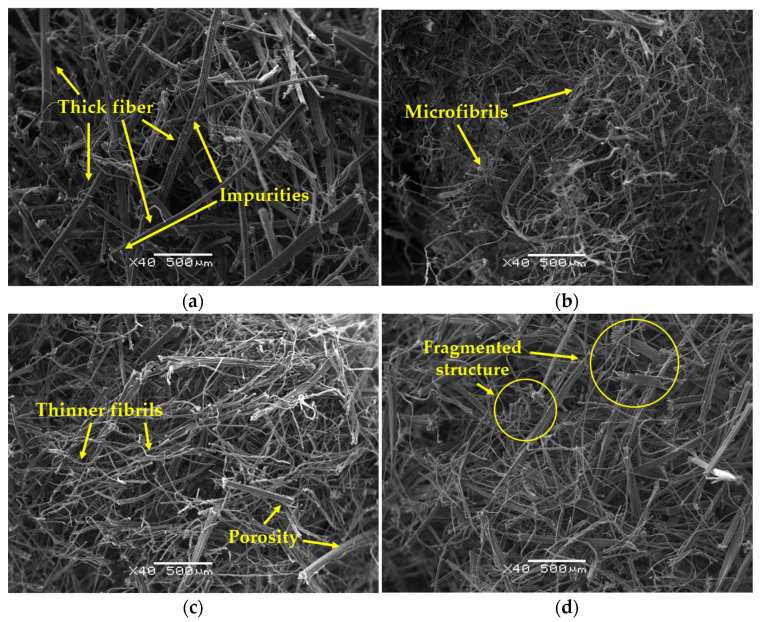
Hemp strands distribution: (**a**) control, (**b**) M2, (**c**) M3, and (**d**) M4.

In order to analyze the influence of the EPTA-based cationization treatment, an SEM analysis was carried out to see the morphology of the hemp samples. Figure 3 shows the hemp strands without treatment and with cationization treatment with the concentrations named in Table 1.

Figure 3a shows untreated hemp fibers with a compact and rigid structure. The fibers are thick with defined edges and adhering impurities. Their morphology is characteristic of unprocessed natural fibers, with a dense and intertwined distribution, suggesting the presence of lignin and other structural components that hold the fibers together. Figure 3b shows a greater separation between the fibers compared to the untreated hemp sample. It is perceived that the action of EPTA has achieved a partial disintegration of the structure, allowing greater exposure of microfibrils. Figure 3c reveals a greater degree of separation of the fibers and thinner fibrils compared to the previous samples. The structure is more exposed and with greater porosity due to greater exposure to EPTA. In Figure 3d, the changes in morphology are significant, the structure is more fragmented, and the fibers show a notable reduction in size. Increasing the temperature of this sample intensified the effects of EPTA, promoting lignin decomposition and greater disintegration of the fibrous network. This is why the texture is more open, which could improve adhesion with PLA.

**Figure 4:**
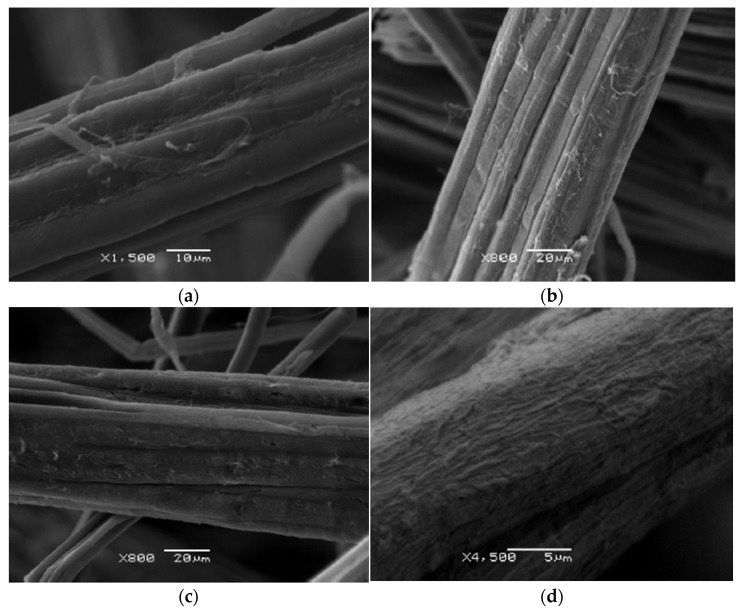
Hemp sample surfaces: (**a**) control, (**b**) M2, (**c**) M3, and (**d**) M4.

The surface of the untreated hemp fiber is relatively smooth, with some superficial cracks and a few exposed fibrillar structures, as shown in Figure 4a. The surface of M2 sample presented a greater roughness compared to the untreated sample, in addition to small fractures as observed in Figure 4b. The M3 sample surface has an even more irregular texture and a greater amount of micro-cracks and detachments, generating a more open and porous structure, according to Figure 4c. The surface of sample M4 is notably the most porous with a highly rough texture, according to Figure 4d. The combination of a higher concentration of EPTA and higher temperature promoted the degradation of lignin and hemicellulose resulting in this eroded surface, which could improve the compatibility of the fiber with polymeric matrices by increasing the contact area and the adhesion capacity [24]. It can be said that the cationization treatment with EPTA gave the expected results in the different concentrations since, in this treatment, the hemp is positively charged to achieve a better dispersion of the fiber [25].

**Figure 5:**
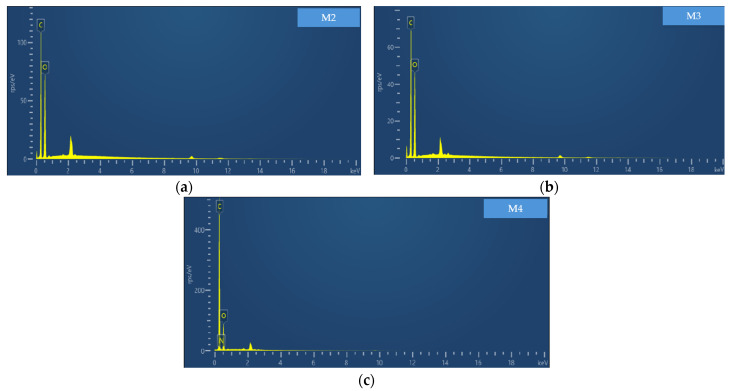
EDS analysis: (**a**) M2, (**b**) M3, and (**c**) M4.

The EDS analysis was used to determine whether the hemp samples contained nitrogen among their elements since this is what provides the positive charges and confirms the cationization reaction with EPTA. This analysis was not performed on sample M1 since the EPTA concentration is too low. Figure 5 shows the results for samples M2, M3, and M4.

In the results of the EDS analysis, sample M2 (see Figure 5a) did not present the nitrogen particle in any of its regions; this is because the concentration of the reagent was not high enough for the nitrogen particle to be present. In sample M3 (see Figure 5b), there was a presence of 0.1%, but since the standard deviation was 0.3, this was discarded. On the other hand, sample M4 (see Figure 5c) presented 1% nitrogen, which can be attributed to the increase in temperature since all the other conditions were exactly the same. Since sample M4 was the one that gave the best results, the tensile specimens with treated hemp were manufactured with this configuration. Table 3 presents a summary of the results obtained from the EDS analysis for each sample.

### 3.2. Tensile Strength

The tensile test results are presented in Table 4 for PLA (PLA only), PH (PLA + untreated hemp), PHG (PLA + untreated hemp and GMA), PHE90 (PLA + treated hemp), and PHGE90 (PLA + treated hemp and GMA) specimens. The σ_t_, ε, and E values correspond to the tensile strength, strain at the end, and elastic modulus, respectively.

**Figure 6:**
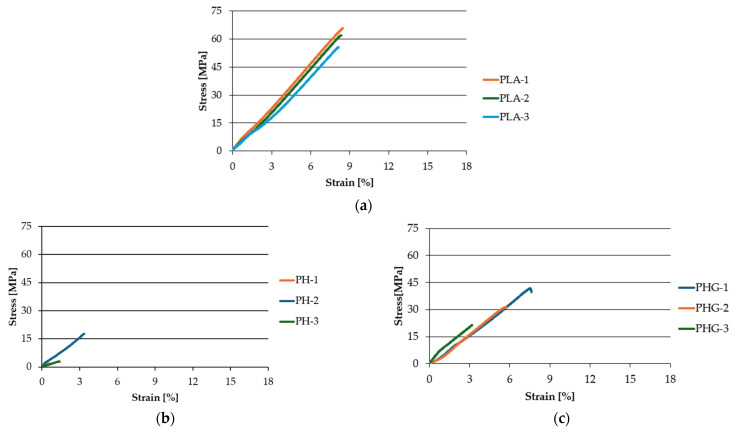

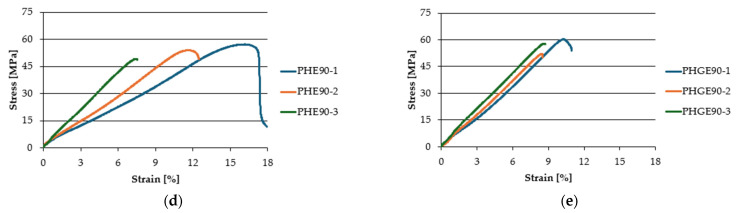
Tensile test curves: (**a**) PLA, (**b**) PH, (**c**) PHG, (**d**) PHE90, and (**e**) PHGE90.

Figure 6 presents the stress-strain curves obtained in the tensile tests performed. PLA exhibited linear elastic behavior up to fracture according to Figure 6a, characteristic of brittle polymers. The combination of untreated hemp with PLA (PH) presented a lower tensile strength and a significant reduction in the maximum strain compared to PLA [19], as observed in Figure 6b. This suggests a poor interaction between the matrix and the fibers, which limits the material’s ability to withstand high loads. On the other hand, the union of untreated hemp with PLA and GMA (PHG) improved the tensile strength compared to the PH samples, indicating that the addition of GMA contributes to improving the adhesion between the polymeric matrix and the untreated hemp fibers. However, the strain continued to be reduced when compared to PLA, as observed in Figure 6c. By combining PLA with treated hemp, a notable increase in tensile strength and an improvement in the deformation capacity before fracture was achieved, as shown in Figure 6d. On the other hand, by combining PLA with treated hemp and GMA, the tensile strength and the deformation capacity before fracture showed increases that only differ from PLA by 7% and 9.5%, respectively. This suggests that the synergy between the treatment of the fibers and the use of GMA optimizes the mechanical properties of the composite [26]. EPTA improved the interfacial adhesion between the hemp fiber and the PLA matrix by modifying the fiber surface, facilitating better dispersion and compatibility with the polymer. This enabled more efficient transfer charges and reduced interfacial defects. On the other hand, GMA acted as a compatibilizing agent by forming chemical bonds between the PLA and the treated hemp, resulting in a stronger material that was less prone to interfacial fractures.

### 3.3. Morphology of the Fractured Surface

**Figure 7:**
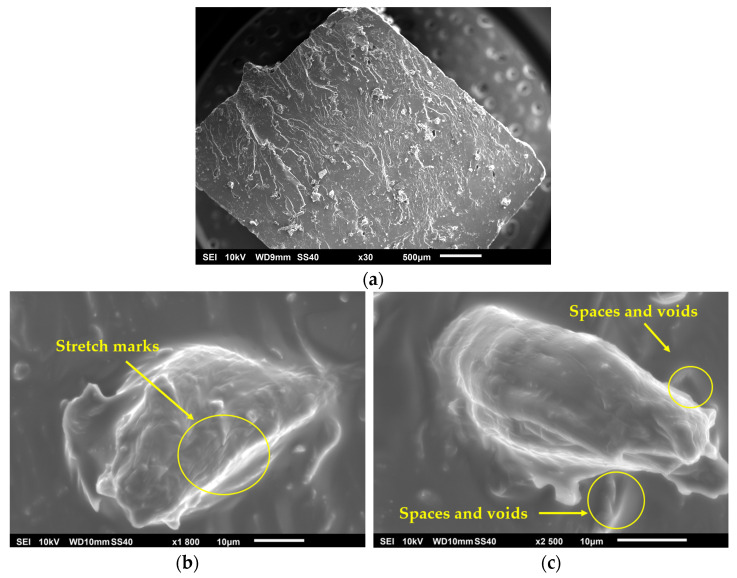
PH specimen: (**a**) 30×, (**b**) 1800×, and (**c**) 2500×.

PLA and hemp present challenges when combined in a composite material, mainly in terms of adhesion and stress transfer, due to the apolar nature of both components. Since both the hemp fibers and the PLA matrix are apolar, the adhesion between the phases is expected to be weak. This can manifest itself in images as separation zones between the fiber and the matrix, indicating poor stress transfer. In Figure 7a, corresponding to the PH specimen, a notable presence of pores is observed, attributed to the separation of the fiber from the matrix without fiber rupture occurring, which contributes negatively by generating stress concentration in the interfacial zones, considered as voids. The lack of adhesion between the fiber and the matrix is evident in the images at 1800× (see Figure 7b) and 2500× (see Figure 7c), where porosities are observed at the interface caused by the displacement of the fiber in the matrix, resulting from the low cohesion between the materials. The type of fracture observed is of a brittle nature, characterized by the presence of striations in the fracture zone, in addition to being an interfacial fracture, as demonstrated by the areas with a high level of porosity caused by the fiber. Likewise, the fibers show signs of not being completely adhered to the matrix, due to voids and spaces around them, which suggests the need for a surface treatment that functionalizes both materials and improves their compatibility.

**Figure 8:**
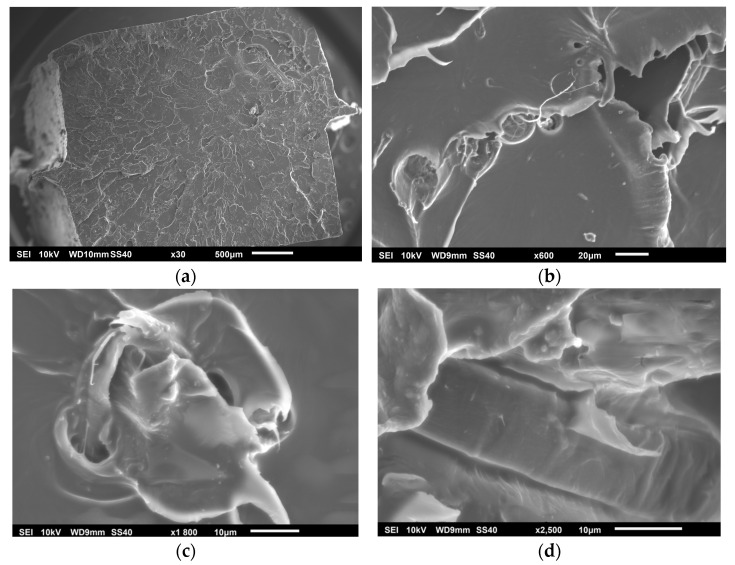
PHGE90 specimen: (**a**) 30×, (**b**) 600× (**c**) 1800× and (**d**) 2500×.

Figure 8a shows the fractured surface of the PHGE90 specimen. An improvement in the adhesion between the hemp fibers and the PLA matrix can be observed. There is a significant reduction in interfacial voids and a better impregnation of the matrix into the hemp fibers, indicating that the chemical treatment has favored a greater compatibility between the phases, creating a stronger interface that allows a better transfer of stresses. Unlike the untreated samples, interfacial fractures are less prevalent. In the areas where the fibers are exposed, a better anchoring in the matrix is noted, suggesting greater resistance to interfacial damage. This improves the overall strength of the composite by delaying damage to the interface. However, there are still areas where fracture continues to occur between the fiber and the matrix, which contributes to the brittle response of the material. The striations observed are indicative of this type of brittle damage. The samples at 1800× (see Figure 8c) and 2500× (see Figure 8d) show better interfacial adhesion compared to the PH specimen in Figure 7, although a lack of adhesion is still detected in the image at 2500×, which is corroborated by the observations at 600× (see Figure 8b).

**Figure 9:**
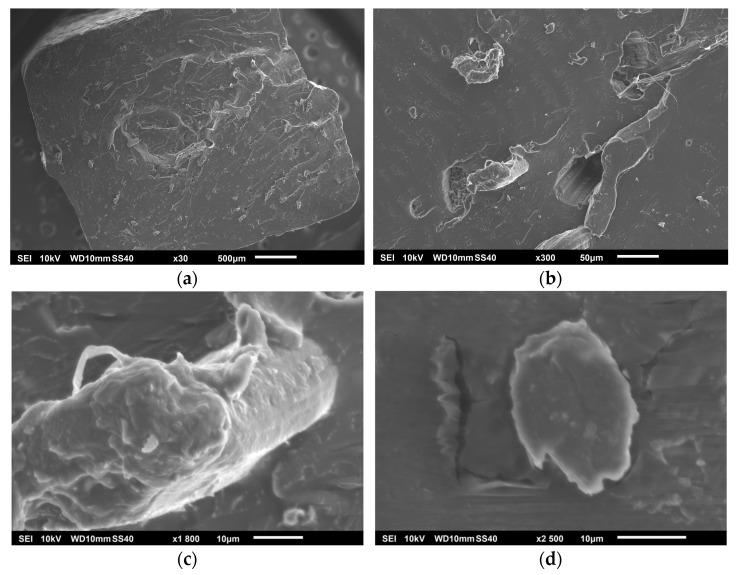
PHG specimen: (**a**) 30×, (**b**) 600× (**c**) 1800×, and (**d**) 2500×.

Figure 9a presents the PHG specimen compared to the previous samples, in which a significant reduction of the interfacial fracture can be seen. Nevertheless, there are still signs of separation between the fiber and the matrix as well as the presence of pores. Brittle behavior remains characteristic of this sample. The adhesion at the interface seems to be more efficient in the PHGE90 (see Figure 8) than in the PHG, which is visible in the areas with low adhesion in the images at 300× (see Figure 9b), 1800× (see Figure 9c), and 2500× (see Figure 9d).

**Figure 10:**
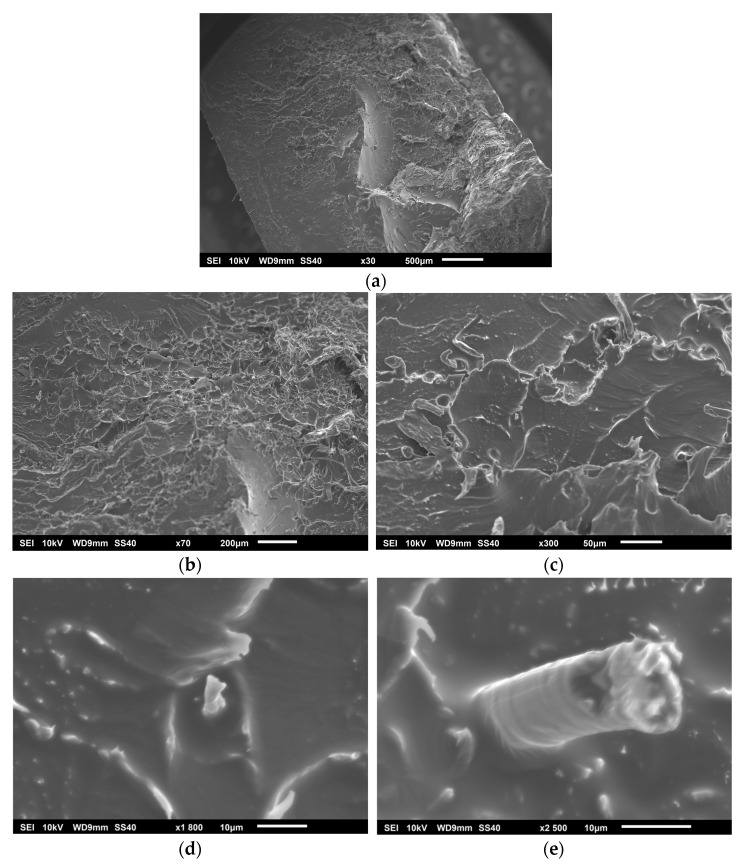
PHE90 specimen: (**a**) 30×, (**b**) 70× (**c**) 300×, (**d**) 1800×, and (**e**) 2500×.

The PHE90 specimen shown in Figure 10a exhibits a completely different behavior from the previous samples. The interfacial adhesion between hemp and PLA is complete, which can be seen in the images at 1800× (see Figure 10d) and 2500× (see Figure 10e). Furthermore, in the specimens in Figure 8 and Figure 9, a fracture perpendicular to the tensile axis (90°) was observed, consistent with a brittle fracture. In contrast, in the sample of Figure 10a, the fracture occurred at 45°, indicating a shear failure, suggesting an improvement in the ductility parameter. This is directly related to the SEM observations, where the adhesion between hemp and PLA is complete, reducing damage propagation and significantly decreasing interfacial fracture. This is evidenced by the images at 30× (see Figure 10a), 70× (see Figure 10b), and 300× (see Figure 10c). These results suggest that the EPTA treatment has been effective, specifically because the ammonium and epoxy groups have generated a significant impact on the interfacial improvement between hemp and PLA. In contrast, the methacrylate group of GMA could have been responsible for not achieving complete adhesion between these materials.

## 4. Conclusions

This study evaluated the effect of incorporating hemp fibers, cationization treatment with (3-chloro-hydroxypropyl) trimethylammonium chloride (EPTA), and functionalization with glycidyl methacrylate (GMA) in improving the mechanical properties of PLA filaments for 3D printing. From the results obtained, the following main conclusions can be drawn.

▪ The addition of 5% untreated hemp in the PLA matrix resulted in an 87.8% reduction in tensile strength, from 61.05 MPa in pure PLA to only 7.47 MPa in the PLA plus untreated hemp blend. A significant reduction in maximum strain was observed, decreasing from 8.32% in pure PLA to only 1.73%, indicating a lower capacity to absorb stress before fracture. This confirms that the interfacial adhesion between PLA and untreated hemp fiber is poor, generating microvoids and structural defects that compromise the strength of the composite.
▪ The cationization of the fiber with EPTA significantly improved compatibility with PLA, resulting in better fiber distribution within the matrix and increased interfacial adhesion. A 615% increase in tensile strength was achieved compared to the PLA and untreated hemp composite, going from 7.47 MPa to 53.40 MPa in the PLA composite sample with treated hemp and EPTA. The elastic modulus increased from 4.32 MPa in the PLA and untreated hemp sample to 4.54 MPa in the sample using EPTA, indicating an improvement in the stiffness of the material. Cationization removed surface impurities and promoted the exposure of microfibrils in the hemp structure, facilitating interaction with the PLA matrix.
▪ The addition of GMA in composites with untreated hemp improved the tensile strength compared to PLA plus untreated hemp, increasing from 7.47 MPa to 31.49 MPa (an increase of 321%). However, the maximum strain was still reduced (5.47%), indicating that although GMA improves adhesion, it is not sufficient on its own to optimize mechanical properties. In the PLA sample with treated hemp and GMA, the best combination of mechanical properties was obtained with a tensile strength of 56.78 MPa, representing only 7% less than pure PLA and 660% more than PLA with untreated fiber. The strain before fracture was 9.11%, 9.5% higher than pure PLA, indicating a more ductile material and less prone to brittle fractures. While the elastic modulus of 6.23 MPa shows better stiffness compared to other formulations. The combination of EPTA treatment and GMA functionalization produced the best mechanical performance. A reduction in the number of interfacial voids and better impregnation of the matrix into the fiber was observed, allowing for more efficient load transfer. Fracture morphology showed that hemp fibers treated with EPTA and functionalized with GMA presented better anchoring within the matrix, reducing defect propagation.
▪ In the samples with untreated hemp, an interfacial fracture was observed with the presence of microvoids at the fiber–matrix interface, confirming weak cohesion between the phases. In the samples with EPTA-treated hemp, a reduction in interfacial defects was evident, with greater integration of the fibers into the PLA matrix, while for the combination of PLA, treated hemp, and GMA, the fracture was more homogeneous and the interfacial adhesion was noticeably better, showing a smaller amount of separation zones between the fiber and the matrix.
▪ EPTA introduced cationic groups to the surface of the hemp fiber, which generated a higher affinity with the PLA matrix. This process improved the dispersion of the fibers within the polymeric matrix, reducing the formation of high-stress concentration zones that can generate premature failures. In addition, the removal of impurities and the exposure of microfibrils in the hemp through cationization allowed a greater contact area with the PLA matrix, favoring the transfer of stress.
▪ GMA acted as a compatibilizer, creating bonds between the treated fiber and the PLA, improving interfacial adhesion. By containing an epoxide group that can react with the hydroxyl groups exposed to the EPTA-treated hemp fiber and with the PLA, it formed covalent bonds. This chemical functionalization reinforced the interaction between the phases, avoiding the formation of voids or defects at the interface, which in turn improved the mechanical strength of the composite material.

The use of PLA reinforced with natural fibers is a sustainable and low-environmental-impact alternative compared to conventional petroleum-derived plastics. Hemp treated with EPTA and functionalized with GMA can be used in FDM 3D printing, offering a balance between mechanical strength and biodegradability.

## Figures and Tables

**Table 1:** Quantities used for reaction.

Specimen	Conditions	Hemp (g)	NaOH (g)	Water (mL)	EPTA (mL)
M1	65 °C and 1500 rpm	1	2.5	47.5	1.66
M2	65 °C and 1500 rpm	1	2.5	47.5	4.17
M3	65 °C and 1500 rpm	1	2.5	47.5	8.34
M4	90 °C and 1500 rpm	1	2.5	47.5	8.34

**Table 2:** Proportions and quantities of mixing material.

Specimen	PLA (%)	Hemp (%)	GMA (mL)	EPTA (mL)	Glycerin (mL)
PLA	100	0	0	0	0
PH	95	5	0	0	4
PHG	95	5	4	0	0
PHE90	95	5	0	8.34	4
PHGE90	95	5	4	8.34	0

**Table 3:** EDS results summary.

	M2			M3			M4		
Element	% Weight	Sigma % Weight	% Atomic	% Weight	Sigma % Weight	% Atomic	% Weight	Sigma % Weight	% Atomic
C	51.55	0.13	58.63	51.44	0.14	58.52	78.81	0.59	83.10
O	48.45	0.13	41.37	48.56	0.14	41.48	20.12	0.24	15.93
N	-	-	-	-	-	-	1.07	0.70	0.97
Total	100.00	-	100.00	100.00	-	100.00	100.00	-	100.00

**Table 4:** Tensile test results.

Specimen	N°	σ_t_ [MPa]	σ_t average_ [MPa]	ε [%]	ε_average_ [%]	E_average_ [MPa]
PLA	1	65.78		8.50		
	2	61.98	61.05	8.35	8.32	7.33
	3	55.38		8.12		
PH	1	1.80		0.48		
	2	17.73	7.47	3.35	1.73	4.32
	3	2.89		1.35		
PHG	1	41.70		7.51		
	2	31.39	31.49	5.71	5.47	5.75
	3	21.37		3.19		
PHE90	1	57.17		16.18		
	2	53.88	53.40	11.67	11.77	4.54
	3	49.16		7.45		
PHGE90	1	60.27		10.25		
	2	52.18	56.78	8.45	9.11	6.23
	3	57.89		6.64		

## Data Availability

The original contributions presented in this study are included in the article. Further inquiries can be directed to the corresponding author.
